# A feasibility study of hyoscine butylbromide (buscopan) to improve image quality of cone beam computed tomography during abdominal/pelvic Stereotactic Ablative Radiotherapy

**DOI:** 10.1259/bjro.20210045

**Published:** 2021-07-29

**Authors:** Finbar Slevin, Matthew Beasley, Jim Zhong, Eleanor Hudson, Richard Speight, John Lilley, Louise J Murray, Ann M Henry

**Affiliations:** 1 Leeds Cancer Centre, Leeds Teaching Hospitals NHS Trust, Beckett Street, Leeds, UK; 2 University of Leeds, Leeds, UK; 3 Department of Radiology, Leeds Teaching Hospitals NHS Trust, Beckett Street, Leeds, UK; 4 Leeds Institute of Clinical Trials Research, University of Leeds, Leeds, UK

## Abstract

**Objectives:**

Cone beam computed tomography (CBCT) is used for image guidance of stereotactic ablative radiotherapy (SABR), but it is susceptible to bowel motion artefacts. This trial evaluated the impact of hyoscine butylbromide (buscopan) on CBCT image quality and its feasibility within a radiotherapy workflow.

**Methods:**

A single-centre feasibility trial (ISRCTN24362767) was performed in patients treated with SABR for abdominal/pelvic oligorecurrence. Buscopan was administered to separate cohorts by intramuscular (IM) or intravenous (i.v.) injection on alternate fractions, providing within-patient control data. 4-point Likert scales were used to assess overall image quality (ranging from excellent to impossible to use) and bowel motion artefact (ranging from none to severe). Feasibility was determined by patient/radiographer questionnaires and toxicity assessment. Descriptive statistics are presented.

**Results:**

16 patients were treated (8 by IM and 8 by i.v. buscopan). The percentage of images of excellent quality with/without buscopan was 47 *vs* 29% for IM buscopan and 65 *vs* 40% for i.v. buscopan. The percentage of images with no bowel motion artefact with/without buscopan was 24.6 *vs* 8.9% for IM buscopan and 25.8 *vs* 7% for i.v. buscopan. Four patients (25%) reported dry mouth. 14 patients (93%) would accept buscopan as routine. 11 radiographers (92%) reported no delay in treatments.

**Conclusions:**

A trend towards improved image quality/reduced bowel motion artefact was observed with IM/i.v. buscopan. Buscopan was well tolerated with limited impact on workflow.

**Advances in knowledge:**

This is the first trial of buscopan within a radiotherapy workflow. It demonstrated a trend to improved image quality and feasibility of use.

## Introduction

Stereotactic ablative radiotherapy (SABR) is increasingly used to treat limited sites of metastatic relapse (so-called oligorecurrence) in the abdomen/pelvis after primary treatment for malignancy.^1–3^ SABR is ultra hypofractionated radiation, delivering large doses per fraction to a highly conformal target volume using steep dose gradients in a small number of fractions. To safely deliver SABR, effective immobilisation and accurate target localisation within millimetre tolerances using image guidance and online correction for interfraction motion and set up errors are required.^4,5^

For linear accelerator-delivered SABR, volumetric image guidance is commonly acquired using cone beam computed tomography (CBCT).^6^ In contrast to diagnostic helical CT, CBCT image projections are typically acquired over at least 1–2 min and are susceptible to motion artefacts (including from bowel) that manifest after reconstruction into volumetric images.^7,8^

In radiology, hyoscine butylbromide (Buscopan^®^ [Sanofi, Reading, UK], herein referred to as buscopan) is routinely used to reduce motion artefacts during MRI of the abdomen and pelvis (among other examinations).^9^

However, administration of anti-peristaltic agents to reduce bowel motion artefacts during radiotherapy has not been previously investigated. In this prospective trial, we evaluated the impact of intramuscular (IM) and intravenous (i.v.) buscopan on CBCT image quality and feasibility of its delivery during an abdominal/pelvic SABR workflow.

## Methods and materials

### Trial design

A single-centre, non-randomised feasibility study was undertaken in Leeds Cancer Centre in patients treated with abdominal/pelvic SABR. The trial was registered on the NIHR Clinical Research Network Portfolio (ID40521) and ISRCTN registry (ISRCTN24362767). Ethical approval from NHS Health Research Authority Research Ethics Committee (referenceXX) was granted March 2019. A CONSORT trials checklist is shown in Supplementary Material 1

### Participants

Participants were identified through Leeds Cancer Centre SABR multidisciplinary meeting. Eligible patients were treated with SABR for oligorecurrent soft tissue/bone metastatic disease in the abdomen/pelvis. Ineligible patients had contraindications to buscopan: severe/recent cardiac disease, tachyarrhythmias, narrow angle glaucoma, myasthenia gravis, mechanical/functional bowel obstruction, obstructive uropathy, porphyria, allergy to buscopan and concomitant administration of anticoagulants (IM buscopan cohort).^9,10^ All participants provided written informed consent.

### Interventions

Buscopan was administered to separate cohorts by IM or i.v. injection on alternate fractions. Each patient therefore provided data without buscopan, acting as a within-person control regarding their individual bowel appearance/motion. Initially, IM buscopan was used since it was considered that this would be more feasible to deliver within a radiotherapy workflow. After review of the first three patients treated with IM buscopan and concern for limited impact on image quality, a substantial amendment to the protocol was made to administer i.v. buscopan.

IM buscopan (20 mg ml^−1^) was administered into the buttock immediately before the patient entered the treatment room. i.v. buscopan (20 mg ml^−1^) was diluted in 10 ml 0.9% sodium chloride and administered over 1 min via a peripherally sited venous cannula, as per institutional protocol. i.v. buscopan was administered once the patient was positioned on the treatment table prior to set-up. The ratio of fractions with/without buscopan was 2:1 and 3:2 for 3 and 5 fraction SABR respectively. Patient involvement in the study finished after their final SABR fraction.

SABR was delivered using a Versa HD^™^ linear accelerator (Elekta AB, Stockholm, Sweden) as 30 Gy in 3–5 fractions on alternate days for soft tissue/non-spinal bone lesions and 24 Gy in 3 fractions for spinal lesions. Patients were positioned supine and immobilised in a BodyFix^®^ vacuum bag (Elekta). CBCTs were acquired using XVI v. 5.04 (Elekta) at baseline (after patient set up), pre-treatment (after target matching and application of shifts in treatment table position) and post-treatment. The following acquisition parameters were used: 120 kV, 20–32 mA, 20–40 ms, 660–1320 projections. Time from injection to each CBCT was recorded. Dietary advice was not provided. For pelvic lesions, scanning was undertaken with empty bladder and rectum.

### Outcomes

The primary end point was improvement in image quality when buscopan was given compared with when it was not given and was assessed using two 4-point Likert scales; an overall image quality scale and a bowel motion artefact scale. The overall image quality scale had the following points: 4 (excellent quality), 3 (satisfactory quality), 2 (poor quality) and 1 (impossible to use). The scale was task-orientated; image quality was scored in the context of matching to the target. The bowel motion artefact scale had the following points: 0 (no artefact), 1 (mild artefact), 2 (moderate artefact) and 3 (severe artefact). Each scale had been internally validated in a retrospective study in which the image quality of CBCT was evaluated in patients previously treated with abdominal/pelvic SABR.^11^

Image quality was evaluated concurrently by MB (senior radiographer) and FS (clinical research fellow) as a consensus score. It was considered that this approach was analogous to the use of image guidance in clinical practice, where pairs of radiographers agree on target matching prior to treatment delivery. Training was performed using images from the previously treated cohort corresponding to each point on the respective Likert scales. Images were viewed in XVI using the following procedure: a random sequence of images per patient was generated using Microsoft Excel 2010 (Microsoft Corporation, Redmond, Washington, USA) and a third person (not scoring the images) loaded each image as per the random sequence. Dates/times were concealed from images to ensure scorers were blinded to whether buscopan was administered. Exploratory analyses of image quality were undertaken for timing of CBCT and whether the treated lesion was pelvic (below level of aortic bifurcation) versus abdominal and soft tissue *vs* bone.

Secondary end points were to demonstrate that administration of IM/i.v. buscopan was feasible within an abdominal/pelvic SABR workflow and was tolerated by patients. These were assessed on the final fraction using a combination of clinician-assessed toxicity (using Common Toxicity Criteria for Adverse Events (CTCAE) v. 5.0) and patient/radiographer questionnaires.^12^ The questionnaires used a 4-point Likert scale with the following points: 1 (not at all), 2 (somewhat), 3 (moderately) and 4 (very much so). For patients, questions primarily concerned their experience of treatment with buscopan and can be seen in Table 1. One radiographer per patient was approached to complete a questionnaire. These questions primarily concerned the impact of buscopan on workflow and can be seen in Table 2. Questionnaire design was based on previously published radiotherapy/MRI patient questionnaires.^13,14^

**Table 1.** Summary of end of treatment patient questionnaire data

*Question*	*Buscopan route of administration*	Median score** (IQR)	*Absolute number of patients indicating a score of 1 (%)*	*Absolute number of patients indicating a score of 2 (%)*	*Absolute number of patients indicating a score of 3 (%)*	*Absolute number of patients indicating a score of 4 (%)*
I understood why the injection was being given	All patients (*n* = 15) IM buscopan (*n* = 8) IV buscopan (*n* = 7)a	4.0 (4.0–4.0) 4.0 (4.0–4.0) 4.0 (4.0–4.0)				15 (100.0%) 8 (100.0%) 7 (100.0%)
Before it was given, I was anxious about having the injection	All patients IM buscopan i.v. buscopan	1.0 (1.0–2.0) 1.5 (1.0–2.3) 1.0 (1–1.5)	9 (60.0%) 4 (50.0%) 5 (71.4%)	4 (26.7%) 2 (25.0%) 6 (85.7%)	1 (6.7%) 1 (12.5%)	1 (6.7%) 1 (12.5%)
I found having the injection frightening	All patients IM buscopan i.v. buscopan	1.0 (1.0–1.0) 1.0 (1.0–1.0) 1.0 (1.0–1.0)	14 (93.3%) 7 (87.5%) 7 (100.0%)			1 (6.7%) 1 (12.5%)
I found the injection painful	All patients IM buscopan i.v. buscopan	1.0 (1.0–2.0) 1.0 (1.0–2.0) 1.0 (1.0–2.0)	9 (60.0%) 5 (62.5%) 4 (57.1%)	5 (33.3%) 2 (25.0%) 3 (42.9%)		1 (6.7%) 1 (12.5%)
I found the injection delayed my treatment	All patients IM buscopan i.v. buscopan	1.0 (1.0–1.0) 1.0 (1.0–1.0) 1.0 (1.0–1.5)	12 (80.0%) 7 (87.5%) 5 (71.4%)	2 (13.3%) 2 (28.6%)		1 (6.7%) 1 (12.5%)
I found the injection gave me side-effects	All patients IM buscopan i.v. buscopan	1.0 (1.0–2.0) 1.0 (1.0–2.0) 1.0 (1.0–1.5)	10 (66.7%) 5 (62.5%) 5 (71.4%)	4 (26.7%) 2 (25.0%) 2 (28.6%)		1 (6.7%) 1 (12.5%)
If I needed treatment again, I would be prepared to have the injection before each fraction	All patients IM buscopan i.v. buscopan	4.0 (4.0–4.0) 4.0 (4.0–4.0) 4.0 (4.0–4.0)			1 (6.7%) 1 (12.5%)	14 (93.3%) 7 (87.5%) 7 (100.0%)

**1=’not at all’, 2=’somewhat’, 3=’moderately’, 4=’very much so’

a Questionnaire data not available for one patient who did not complete radiotherapy as planned due to acute admission

**Table 2.** Summary of end of treatment radiographer questionnaire data

*Question*	*Buscopan route of administration*	Median score** (IQR)	*Absolute number of radiographers indicating a score of 1 (%)*	*Absolute number of radiographers indicating a score of 2 (%)*	*Absolute number of radiographers indicating a score of 3 (%)*	*Absolute number of radiographers indicating a score of 4 (%)*
I understood why buscopan was being given	All patients (*n* = 12) a IM buscopan (*n* = 6) i.v. buscopan (*n* = 6)	3.3 (3.0–4.0) 3.8 (3.1–4.0) 3.0 (3.0–3.8)			6 (50.0%) 3 (50.0%) 3 (50.0%)	6 (50.0%) 4 (66.7%) 2 (33.3%)
I had to wait for someone to attend to administer buscopan	All patients IM buscopan i.v. buscopan	1.0 (1.0–2.1) 1.0 (1.0–1.0) 1.5 (1.0–2.8)	8 (66.7%) 5 (83.3%) 3 (50.0%)	1 (8.3%) 1 (16.7%)	2 (16.7%) 1 (16.7%) 1 (16.7%)	1 (8.3%) 1 (16.7%)
Administration of buscopan delayed the patient’s treatment	All patients IM buscopan i.v. buscopan	1.0 (1.0–1.0) 1.0 (1.0–1.0) 1.0 (1.0–1.0)	11 (91.7%) 6 (100.0%) 5 (83.3%)		1 (8.3%) 1 (16.7%)	
Administration of buscopan appeared to be painful for the patient	All patients IM buscopan i.v. buscopan	1.0 (1.0–1.0) 1.0 (1.0–1.0) 1.0 (1.0–1.0)	12 (100.0%) 6 (100.0%) 6 (100.0%)			
Buscopan appeared to give the patient side-effects	All patients IM buscopan i.v. buscopan	1.0 (1.0–1.0) 1.0 (1.0–1.0) 1.0 (1.0–1.0)	12 (100.0%) 6 (100.0%) 6 (100.0%)			
I thought that image quality was better when buscopan was given	All patients IM buscopan i.v. buscopan	2.3 (2.0–3.0) 2.3 (2.0–2.9) 2.5 (2.0–3.0)	1 (8.3%) 1 (16.7%)	5 (41.7%) 3 (50.0%) 2 (33.3%)	4 (33.3%) 2 (33.3%) 1 (16.7%)	1 (8.3%) 1 (16.7%)
I would be prepared for buscopan to be given routinely for abdominal/pelvic SABR treatments	All patients IM buscopan i.v. buscopan	4.0 (3.0–4.0) 3.5 (3.0–4.0) 4.0 (3.3–4.0)			5 (41.7%) 3 (50.0%) 2 (33.3%)	7 58.3%) 3 (50.0%) 4 (66.7%)

SABR, stereotactic ablative radiotherapy

**1=’not at all’, 2=’somewhat’, 3=’moderately’, 4=’very much so’

a Radiographer questionnaire data not available for four patients

### Statistics

This feasibility study was not designed to demonstrate statistical significance; therefore, there was no formal sample size calculation.^15^ Given each patient received 3 scans per 3/5 fractions, a sample of 16 patients provided between 144 and 240 images and was considered sufficient information. Each patient provided data with/without buscopan, acting as a within-person control. However, pairing of data to utilise standard statistical paired tests was infeasible, with no clear pairing and violation of the assumption of independent samples due to pairing across multiple scan/time points for the same patient. More sophisticated approaches, that account for the complex data structure, such as mixed modelling, were considered unsuitable for the number of patients. Therefore, descriptive statistics are presented including median and interquartile range (IQR). To account for differences in numbers of images with/without buscopan and intrapatient correlation in bowel motion, image quality scores were summarised per patient prior to being summarised for the whole cohort.^16,17^

## Results

### Participants

**Figure 1.**
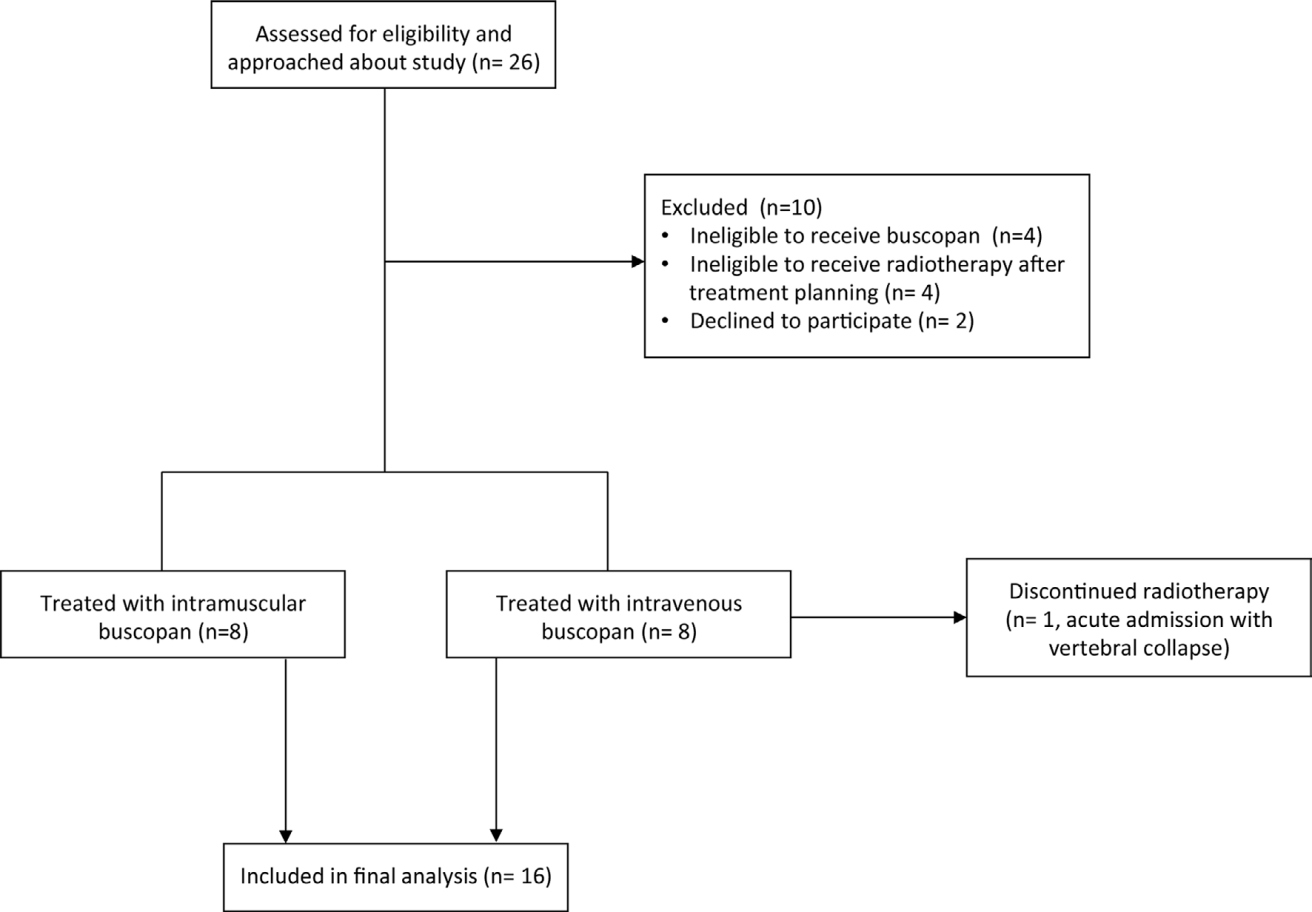
Flow diagram showing numbers of participants approached for the study, numbers of patients excluded/recruited and numbers of patients who completed the study.

The trial schema is shown in Figure 1. 10 patients were excluded. 16 patients commenced treatment (8 in the IM cohort and 8 in the i.v. cohort); of these, one patient in the i.v. cohort experienced vertebral collapse and SABR was stopped early. The first patient was recruited September 2019 and the final patient completed treatment January 2021. Study recruitment was paused for 6 months from March to July 2020 during the Covid-19 pandemic.

Baseline demographic and clinical characteristics of the patients are shown in Supplementary Material 1. 11 patients received 5-fraction SABR and 5 received 3 fractions. Eight lesions were in soft tissue and eight were in bone. 10 lesions were pelvic and 6 were abdominal.

### Primary endpoint

**Figure 2.**
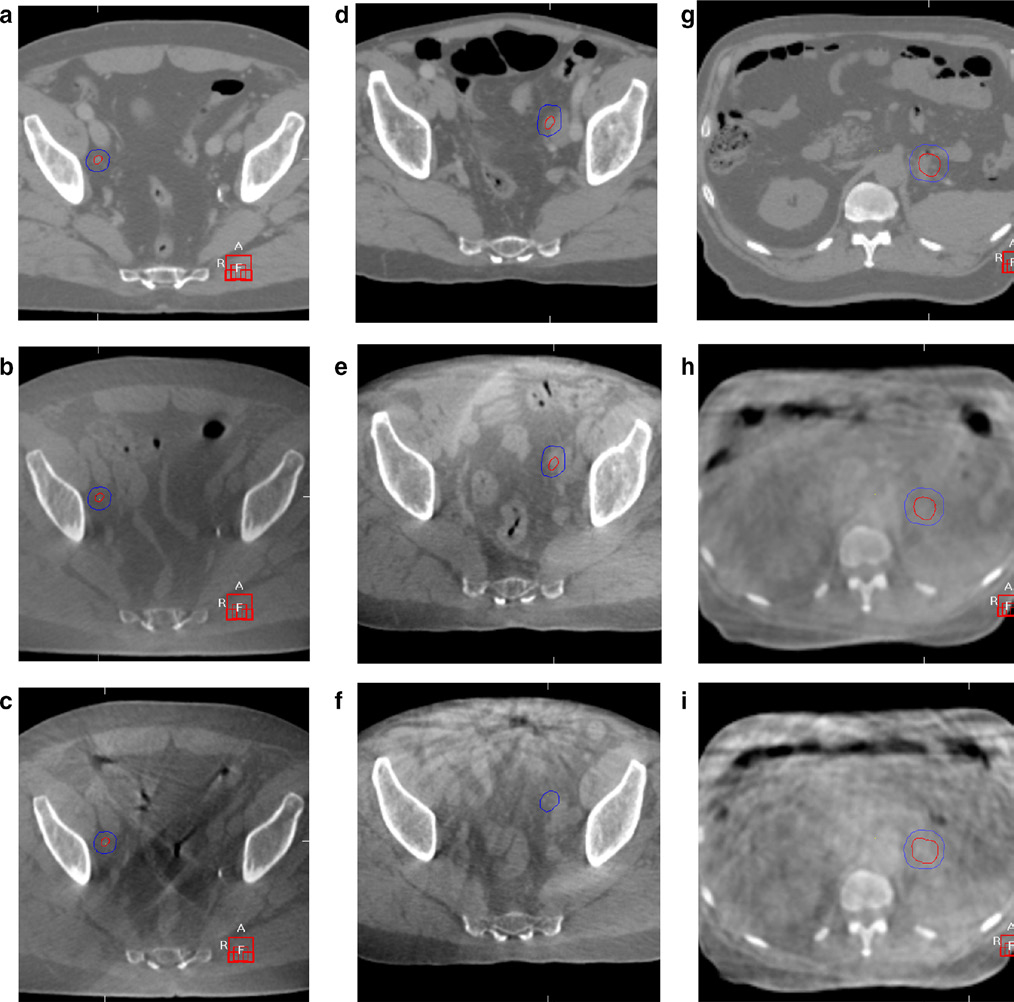
Planning CT and CBCT images with/without IM and i.v. buscopan for three patients. GTV and PTV are shown in each image. CBCT, cone beam CT; GTV, gross tumour volume; PTV, planning target volume.

Figure 2 illustrates the impact of IM and i.v. buscopan on bowel motion artefact in CBCT images.

16 patients were included in the image quality analyses (8 in IM cohort and 8 in i.v. cohort); 127 images with buscopan (65 and 62 in IM and i.v. cohorts respectively) and 88 without buscopan (45 and 43 in IM and i.v. cohorts respectively). One image (without buscopan) in the i.v. cohort was excluded because of scan failure.

**Figure 3.**
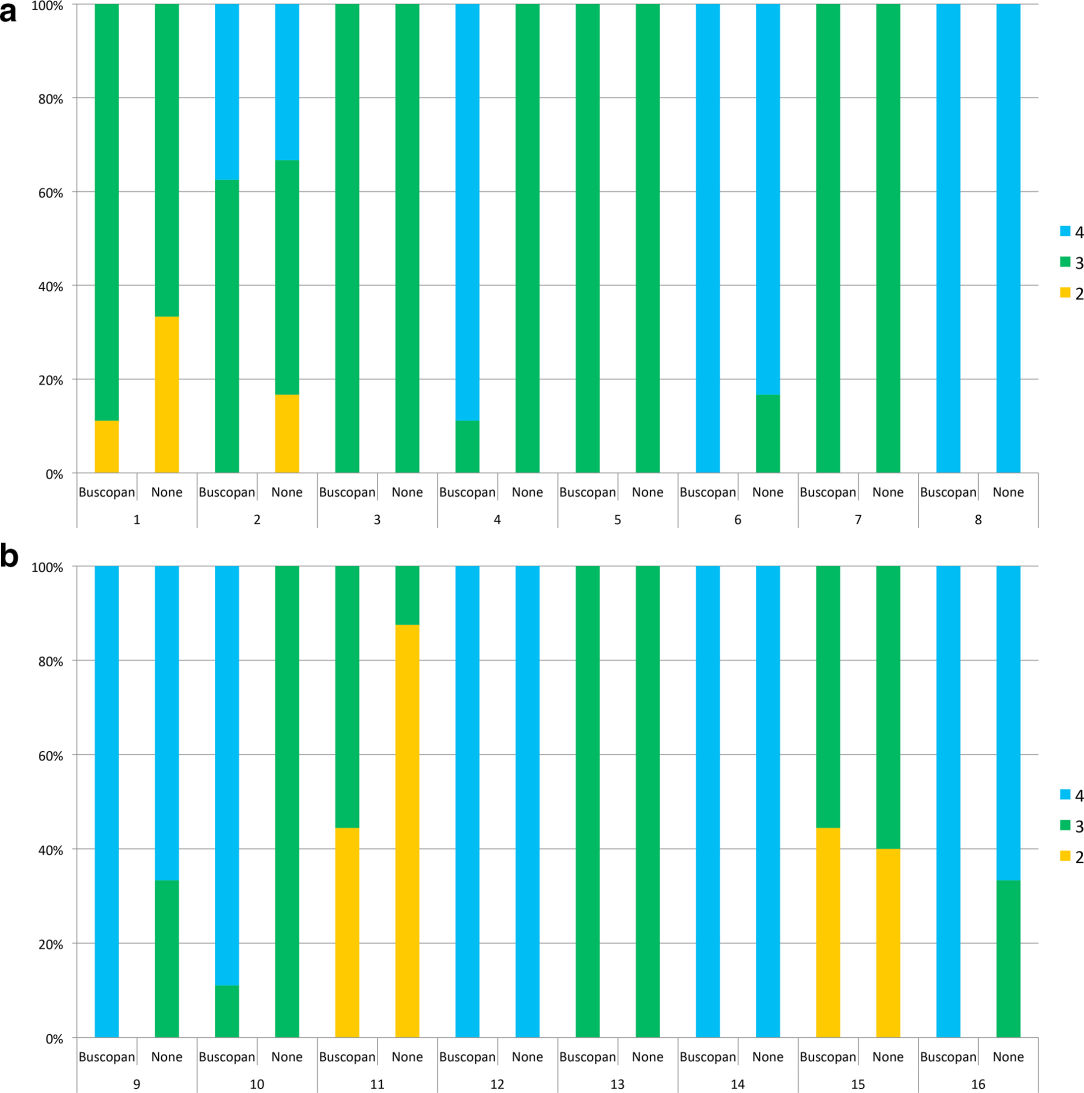
In Figure 3A–B, overall image quality scores are shown with/without buscopan for each patient treated with IM buscopan (A, patients 1–8) and i.v. buscopan (B, patients 9–16). 4 = excellent image quality, 3 = satisfactory image quality and 2 = poor image quality. IM, intramuscular.

For patients who received IM buscopan, the percentage of images of excellent quality with/without buscopan was 47 *vs* 29%. For patients who received i.v. buscopan, the percentage of images of excellent quality with/without buscopan was 65 *vs* 40%. A summary of overall image quality and the proportion of images corresponding to each point on the scale is shown in Table 3. The proportion of scores per patient is illustrated in Figure 3. Individual patient data is shown in Supplementary Material 1.

**Table 3.** Summary of overall image quality scores and proportion of individual scores by receipt of buscopan

*Image type*	*Number of patients*	*Number of images*	Median Likert scale score* (IQR)	*Absolute number of images with score of 4 (%)*	*Absolute number of images with score of 3 (%)*	*Absolute number of images with score of 2 (%)*	*Absolute number of images with score of 1 (%)*
**Images with IM buscopan**	**8**	**65**	**3.0 (3.0–4.0**)	**29** (**44.6%**)	**35** (**53.8%**)	**1** (**1.5%**)	**0**
Images without IM buscopan	8	45	3.0 (3.0–3.3)	13 (28.9%)	29 (64.4%)	3 (6.7%)	0
**Images with i.v. buscopan**	**8**	**62**	**4.0 (3.0–4.0**)	**40** (**64.5%**)	**14** (**22.6%**)	**8** (**12.9%**)	**0**
Images without i.v. buscopan	8	43	3.5 (3.0–4.0)	17 (39.5%)	17 (39.5%)	9 (20.9%)	0
**All images with buscopan**	**16**	**127**	**3.5 (3.0–4.0**)	**70** (**55.1%**)	**49** (**38.6%**)	**9** (**7.1%**)	**0**
All images without buscopan	16	88	3.0 (3.0–4.0)	30 (34.1%)	46 (52.3%)	12 (13.6%)	0

IM, intra muscular; IQR, inter quartile range; IV, intravenous; min, minimum; max, maximum

*4=excellent quality, 3 = satisfactory, 2 = poor, 1 = impossible to use

**Figure 4.**
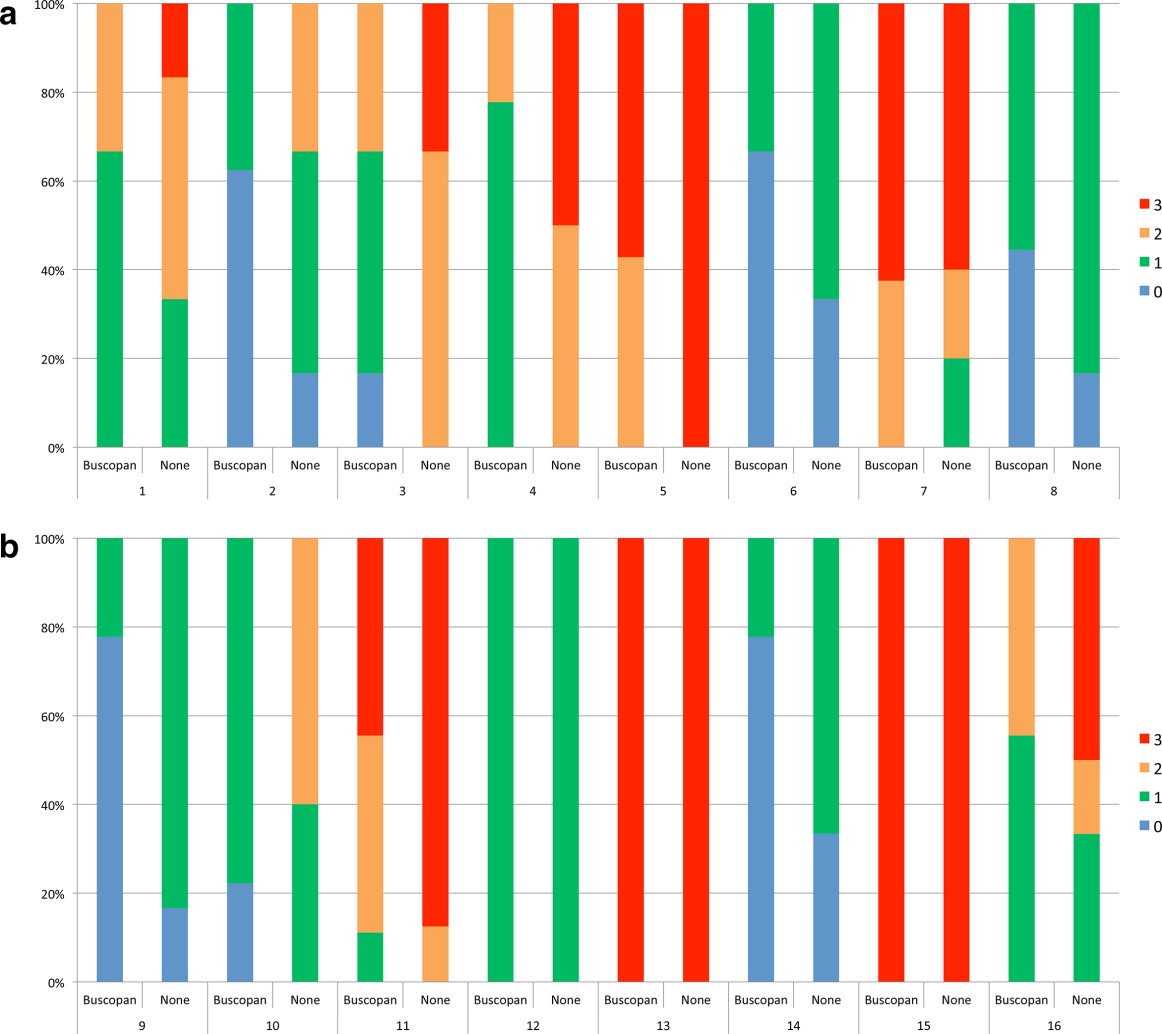
In Figure 4A–B, bowel motion artefact scores are shown with/without buscopan for each patient treated with IM buscopan (A, patients 1–8) and i.v. buscopan (B, patients 9–16). 0 = no artefact, 1 = mild artefact, 2 = moderate artefact and 3 = severe artefact.

For patients who received IM buscopan, the percentage of images with no bowel motion artefact with/without buscopan was 24.6 *vs* 8.9%. For patients who received i.v. buscopan, the percentage of images of excellent quality with/without buscopan was 25.8 *vs* 7%. A summary of bowel motion artefact and the proportion of images corresponding to each point on the scale is shown in Table 4. The proportion of scores per patient is illustrated in Figure 3. Individual patient data are shown in Supplementary Material 1.

**Table 4.** Summary of bowel motion artefact scores and proportion of individual scores by receipt of buscopan

*Image type*	*Number of patients*	*Number of images*	Median bowel motion artefact scale score* (IQR)	*Absolute number of images with score of 0 (%)*	*Absolute number of images with score of 1 (%)*	*Absolute number of images with score of 2 (%)*	*Absolute number of images with score of 3 (%)*
**Images with IM buscopan**	**8**	**65**	**1.0 (0.8–1.5**)	**16** (**24.6%**)	**27** (**41.5%**)	**13** (**20%**)	**9** (**13.8%**)
Images without IM buscopan	8	45	2.0 (1.0–2.6)	4 (8.9%)	15 (33.3%)	11 (24.4%)	15 (33.3%)
**Images with i.v. buscopan**	**8**	**62**	**1.0 (0.8–2.3**)	**16** (**25.8%**)	**22** (**35.5%**)	**8** (**12.9%**)	**16** (**25.8%**)
Images without i.v. buscopan	8	43	2.3 (1.0–3.0)	3 (7%)	16 (37.2%)	5 (11.6%)	19 (44.2%)
**All images with buscopan**	**16**	**127**	**1.0 (0.8–2.3**)	**32** (**25.2%**)	**50** (**39.4%**)	**21** (**16.5%**)	**25** (**19.7%**)
All images without buscopan	16	88	2.0 (1.0–3.0)	7 (8%)	31 (35.2%)	16 (18.2%)	34 (38.6%)

IM, intramuscular; IQR, inter quartile range; IV, intravenous

*0=no bowel motion artefact, 1 = mild bowel motion artefact, 2 = moderate bowel motion artefact, 3 = severe bowel motion artefact

Summaries of image quality by timing of CBCT and for pelvic *vs* abdominal and soft tissue versus bone lesions are shown in Supplementary Material 1.

Median time (IQR) from injection to baseline, pre-treatment and post-treatment imaging was 10 min (7–11) and 7 min (5–8), 14 min (13–17) and 12 min (11–15) and 21 min (17–26) and 20 min (17–26) for IM and i.v. buscopan respectively.

### Secondary end points

A summary of patient questionnaire data for 15 patients is shown in Table 1. Questionnaire data were not available for the patient who did not complete SABR as planned. 14 patients (93%) who completed questionnaires would accept buscopan prior to routine SABR treatment.

A summary of radiographer questionnaire data for 12 radiographers is shown in Table 2. Questionnaires were offered to 16 radiographers and 12 accepted. 11 radiographers (92%) reported no delay in patients’ treatments as a result of buscopan.

### Toxicity

A summary of acute toxicities is shown in Table 5. No ≥Grade 3 toxicities were observed.

**Table 5.** Summary of acute toxicity data

*Acute toxicity*	*CTCAE gradea*	*Total number of patients (% of 16)b*	*Number of patients treated with IM buscopan (% of 8)*	*Number of patients treated with i.v. buscopan (% of 8)*
None		6 (37.5%)	4 (50.0%)	2 (25.0%)
Dry mouth	1	3 (18.8%)	1 (12.5%)	2 (25.0%)
	2	1 (6.3%)	1 (12.5%)	
Injection site discomfort/bruising	1	2 (12.5%)	1 (12.5%)	1 (12.5%)
Cannula removal discomfort	1	1 (6.3%)		1 (12.5%)
Abdominal pain	1	1 (6.3%)		1 (12.5%)
Diarrhoea	1	4 (25.0%)	2 (25.0%)	2 (25.0%)

CTCAE, Common Toxicity Criteria for Adverse Events; IM, intramuscular; IV, intravenous

a Toxicity graded as per Common Toxicity Criteria for Adverse Events (CTCAE) version 5.0

b Total exceeds 100% since some patients reported more than one toxicity

## Discussion

This is the first study to evaluate the impact of anti peristaltic agents in radiotherapy/on CBCT image quality. We observed a trend to improved overall image quality when buscopan was given. The percentage difference in image quality without buscopan between the IM and i.v. cohorts demonstrates considerable inter patient variation, possibly as a result of individual bowel appearance/motion and this validates the approach of using patients as their own control. There was also a trend to reduced bowel motion artefacts with buscopan. Since the study was not powered to detect a statistically significant improvement in image quality, these findings should be considered as a signal of the anti peristaltic effect of buscopan.

The administration of buscopan appeared to be feasible. In general, IM and i.v. buscopan was well tolerated by patients as evidenced by toxicity assessment/questionnaire responses. Dry mouth and injection site discomfort were related to buscopan, with abdominal discomfort/diarrhoea more likely due to SABR. Previous prospective/randomised studies of buscopan during abdominal/pelvic MRI differ in the toxicities reported. Some reported blurred vision in up to 20% of participants, with dry mouth (63%), warmth (20%), dizziness (11%) and palpitations (6%) also described in a study by Johnson et al.^18–21^ Rate of administration of i.v. buscopan was not described in Johnson et al, but it is possible that administration over 1 min minimised toxicity in our i.v. cohort.^22^ Other studies reported no toxicity, although Johnson et al used patient questionnaires to assess toxicity.^20,23–26^ Radiographer questionnaire responses suggested that buscopan did not negatively impact on workflow. This is despite no specific slot for cannulation/administration of buscopan having been booked for patients, which would likely aid patient flow in routine practice.

Several previous prospective and two randomised radiology studies have evaluated the impact of buscopan on image quality for MRI of the abdomen and pelvis.^21,23–29^ Heterogeneity exists between these studies for the route of buscopan administration, the method of image analysis (qualitative Likert-type scales versus measurement of image noise) and whether bowel is evaluated or another organ/lesion. Nevertheless, in prospective studies administration of IM/i.v. buscopan was associated with significantly improved image quality, reduced bowel motion artefacts and improved organ/lesion identification.^21,25–29^ In two randomised studies (which quantified image noise with/without buscopan), a significant reduction in bowel artefact noise was observed when buscopan was administered.^23,24^

The magnitude of improvement in image quality in our study was modest. However, accepting differences in measurement between studies the improvements we observed are comparable to the differences in overall image quality/bowel motion artefact of approximately 0.5–1.0 on 5-point scales reported in prospective studies of buscopan in MRI abdomen/pelvis.^21,26,27^ In contrast to radiology where the clarity of lesion visualisation may be of critical diagnostic importance, the clinical benefits of the improvements in image quality/reduced bowel motion artefacts on CBCT that we observed are less easy to define. The percentage of images scored as poor for overall quality with buscopan was almost half that without. However, all of the patients in the study proceeded with treatment regardless of the quality of their images and no image was scored as impossible to use, which suggests that for CBCT-guided SABR poor quality may be good enough. The published MRI data, combined with the feasibility that we demonstrated of delivering buscopan within a SABR workflow, suggest that a useful application of buscopan could be for MR linac-delivered SABR. A concern with the delivery of ablative doses in the abdomen and pelvis is the risk of toxicity, especially concerning bowel. The greater soft tissue visualisation afforded by MRI compared with CBCT and online reoptimisation may provide the opportunity to adapt the delivered dose based on the daily position of adjacent OARs such as bowel.^30,31^ This approach would require confidence in clearly delineating both the target and OARs. Buscopan could therefore be an important adjunct to improve the quality of images for bowel delineation, for which deformable image registration/autocontouring strategies remain under investigation.^32^ Ease of target/bowel visualisation, time taken for delineation and the extent/dosimetric consequences of intrafraction bowel motion with/without buscopan could be endpoints measured within a future trial.

Although our study was not designed to compare IM and i.v. buscopan, we observed similar image quality scores by both administration routes, with slightly improved summary data for i.v. buscopan. We did not observe any trends in image quality based on the timing of CBCT. Limited data exist concerning the onset and duration of anti-peristaltic effects by different routes of administration, but they approximately support action of buscopan within our time window between first and last CBCT. Previous studies of buscopan in small bowel cine MRI reported approximate onset of action of i.v. buscopan and IM buscopan of <90 sec and 5 min respectively.^19,33^ Mean duration of action was reported to be 21–23 min and approximately 18 min for i.v. and IM buscopan respectively. Large variations between participants in the onset, extent and duration of response were observed in these small studies, especially concerning IM buscopan. It was speculated that this could be due to slower/less reliable absorption of drug via the IM route.^33^ However, other studies that evaluated the impact of buscopan on image quality of abdominal MRI administered IM buscopan around 20 min prior to the examination with persisting anti-peristaltic effects.^34,35^ All of this means that, while the MRI data support greater rapidity/reliability of anti-peristaltic effect with i.v. buscopan, it might still be possible to observe a benefit in reduction in CBCT bowel motion artefact with IM buscopan if cannulation/administration of i.v. buscopan is not practical.

A further consideration concerns the location of the treated lesion. In this study, 39% of images without buscopan were scored as containing severe bowel motion artefacts, although only 14% of images were considered to be of overall poor quality. This discrepancy may be related to the scoring process we used, where overall image quality was assessed in the context of the ability to match to the target. 50% of lesions occurred in bone, where the automatic registration between planning CT and CBCT typically works well.^36^ These patients were included where bowel was close to the lesion but it meant that an image could be scored as being of overall satisfactory quality despite the presence of severe bowel motion artefact, and therefore buscopan may have less impact on matching for bone lesions. We also observed inferior image quality with/without buscopan for abdominal lesions compared with pelvic lesions (median bowel motion artefact score 3 *vs* 1), which is likely secondary to the influence of respiratory motion. Few upper abdominal soft tissue lesions were treated during the study period but, for these, the application of motion management strategies such as breath-hold/respiratory gating in combination with buscopan could be investigated.^37,38^

This study has several limitations. The number of patients was small and there was no statistical comparison of image quality with/without buscopan. Our methods of image assessment were inherently subjective and our use of a consensus score meant that the results could have been over influenced by one of the scorers. However, we used example images for training and similar Likert scales were used in many of the prospective radiology studies of buscopan in MRI abdomen/pelvis. An alternative approach of quantitative assessment of image noise may be influenced by patient motion/variations in acquisition of regions of interest for measurement.^20,21,23,25–27,29^ Other factors, such as soft tissue contrast, may influence CBCT image quality but we did not attempt to incorporated this into our qualitative image assessment.^39^ Other methods of improving CBCT image quality by reduction of image noise and motion artefacts exist, such as dual-energy CT, anti scatter grids, beam filters and reconstruction algorithms.^40,41^ However, CBCT systems with advanced capabilities may not yet be widely implemented in radiotherapy departments, meaning that there remains a value in investigating the impact of anti peristaltic agents on bowel motion artefacts. Some data were missing, which may have influenced our conclusions regarding the feasibility of IM/i.v. buscopan; toxicity assessment/patient questionnaires from one patient who did not complete SABR and radiographer questionnaires from four patients.

## Conclusion

A trend to improved image quality was observed with buscopan and its use in a SABR workflow appears to be feasible. The clinical benefits of buscopan should be investigated and might be best evaluated as part of an MR-guided adaptive SABR workflow.

In Figure 2A–C and a planning CT for a right external iliac nodal metastasis is shown in A, CBCT with IM buscopan in B and CBCT without IM buscopan in C. Reduced streak artefact from bowel gas is apparent in B compared with C.

In Figure 2D–F and a planning CT for a left external iliac nodal metastasis is shown in D, CBCT with i.v. buscopan in E and CBCT without i.v. buscopan in F. Reduced streak artefact from bowel gas is apparent in E compared with F.

In Figure 2G–I and a planning CT for a pancreatic tail metastasis is shown in G, CBCT with i.v. buscopan is shown in H and CBCT without i.v. buscopan is shown in I. Despite some apparent reduction in bowel motion artefact in H compared with I, persistent artefact is shown in H and is likely related to respiratory motion.
